# Supplementary figures and images for: Cytogenetic analyses in Trinomys (Echimyidae, Rodentia), with description of new karyotypes
Source: PeerJ. 2018 Jul 31;6:e5316. doi: 10.7717/peerj.5316 (PMC6074804; doi:10.7717/peerj.5316)

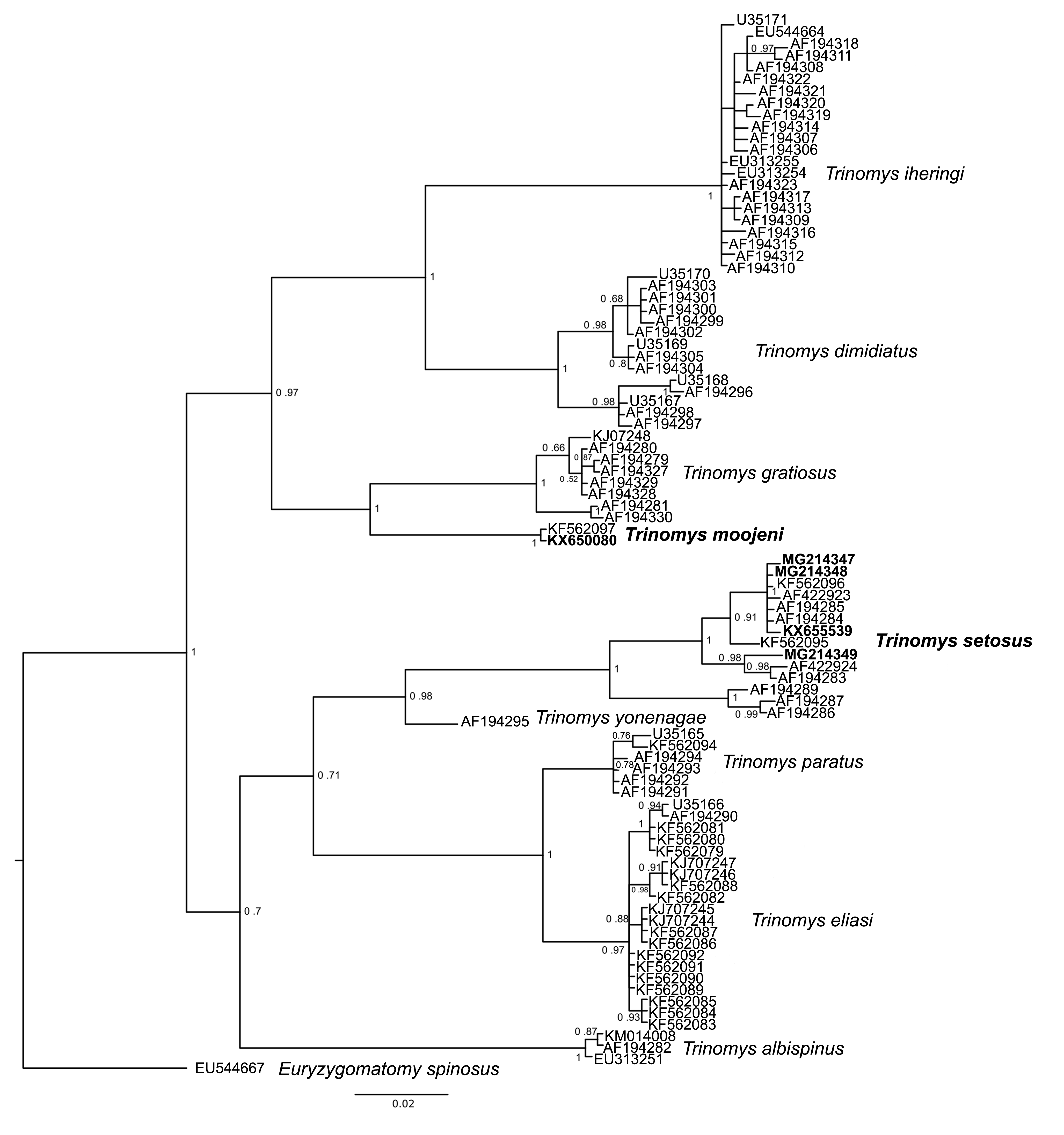

Supplement: Figure S1 — Euryzygomatomys spinosus was used as outgroup. Numbers indicate Bayesian posterior probabilities ≥0.95. Specimens included in this study are in bold. Scale bar represents the number of substitutions per site. [file peerj-06-5316-s001.png]

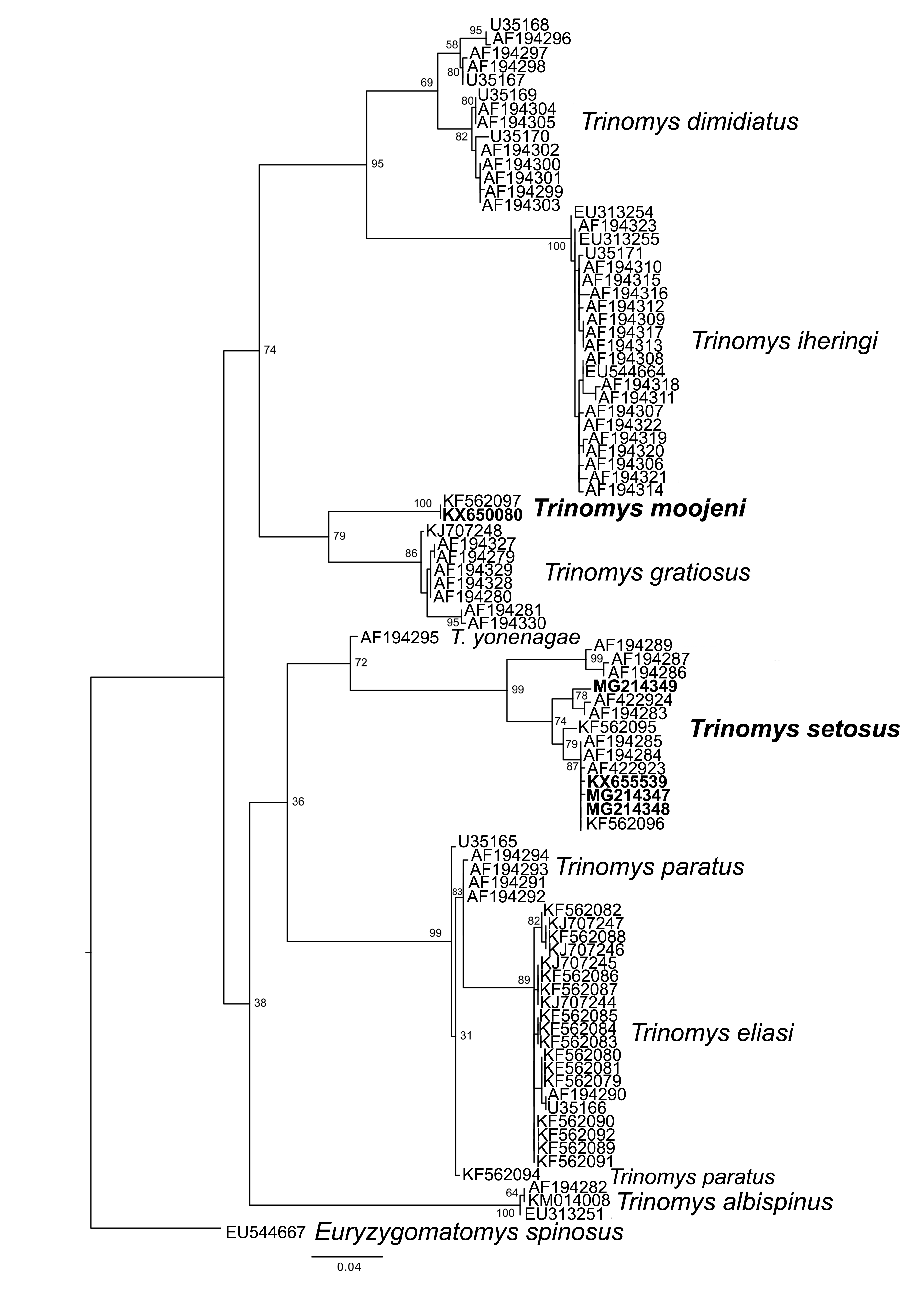

Supplement: Figure S2 — Numbers represent bootstrap support. Specimens included in this study are in bold. Scale bar indicates the number of substitutions per site. [file peerj-06-5316-s002.png]

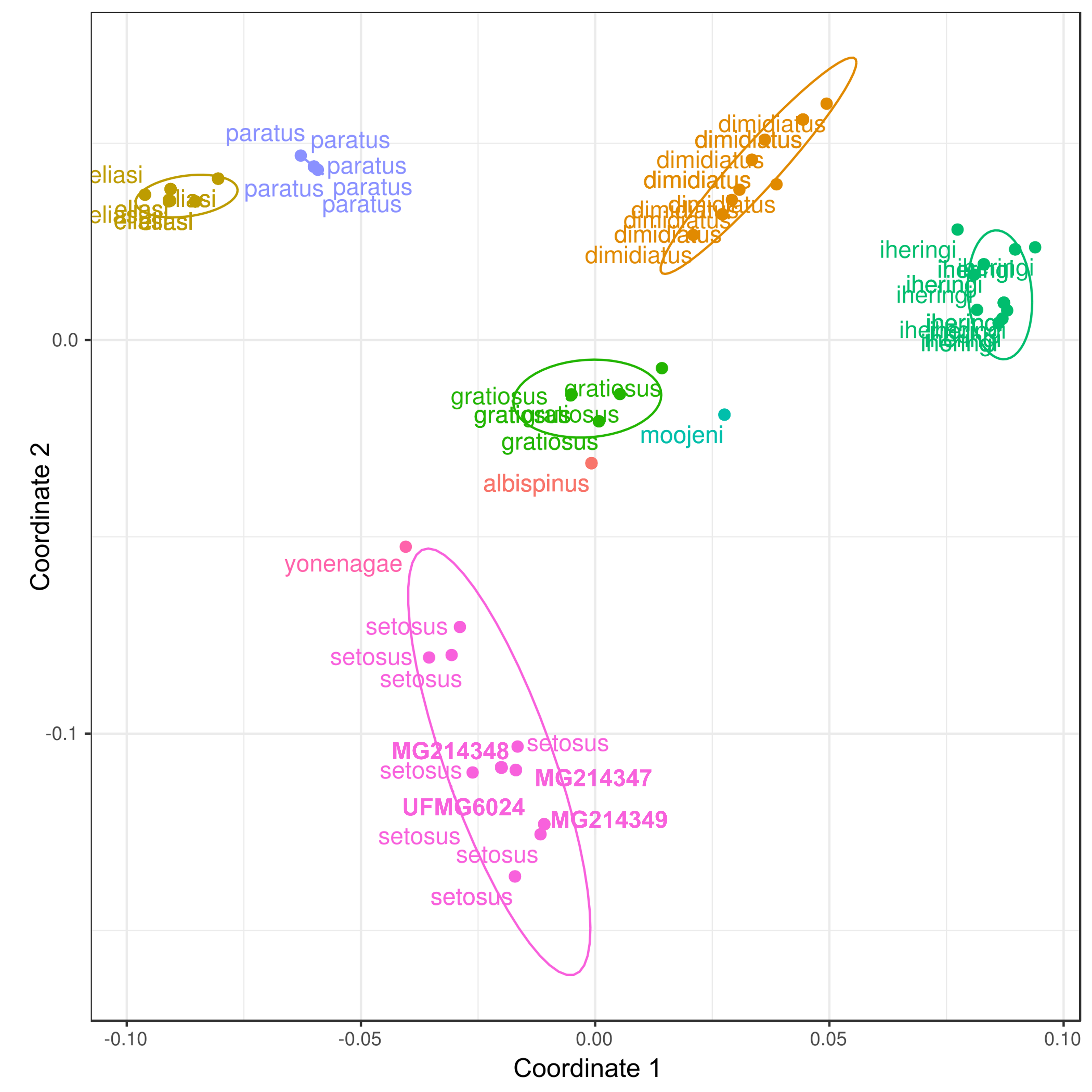

Supplement: Figure S3 — Two-dimensional PCoA ordination diagram of Trinomys species, showing the similarity (or dissimilarity) between individuals. The specimens included in the present study are indicated by their deposit numbers. [file peerj-06-5316-s003.png]

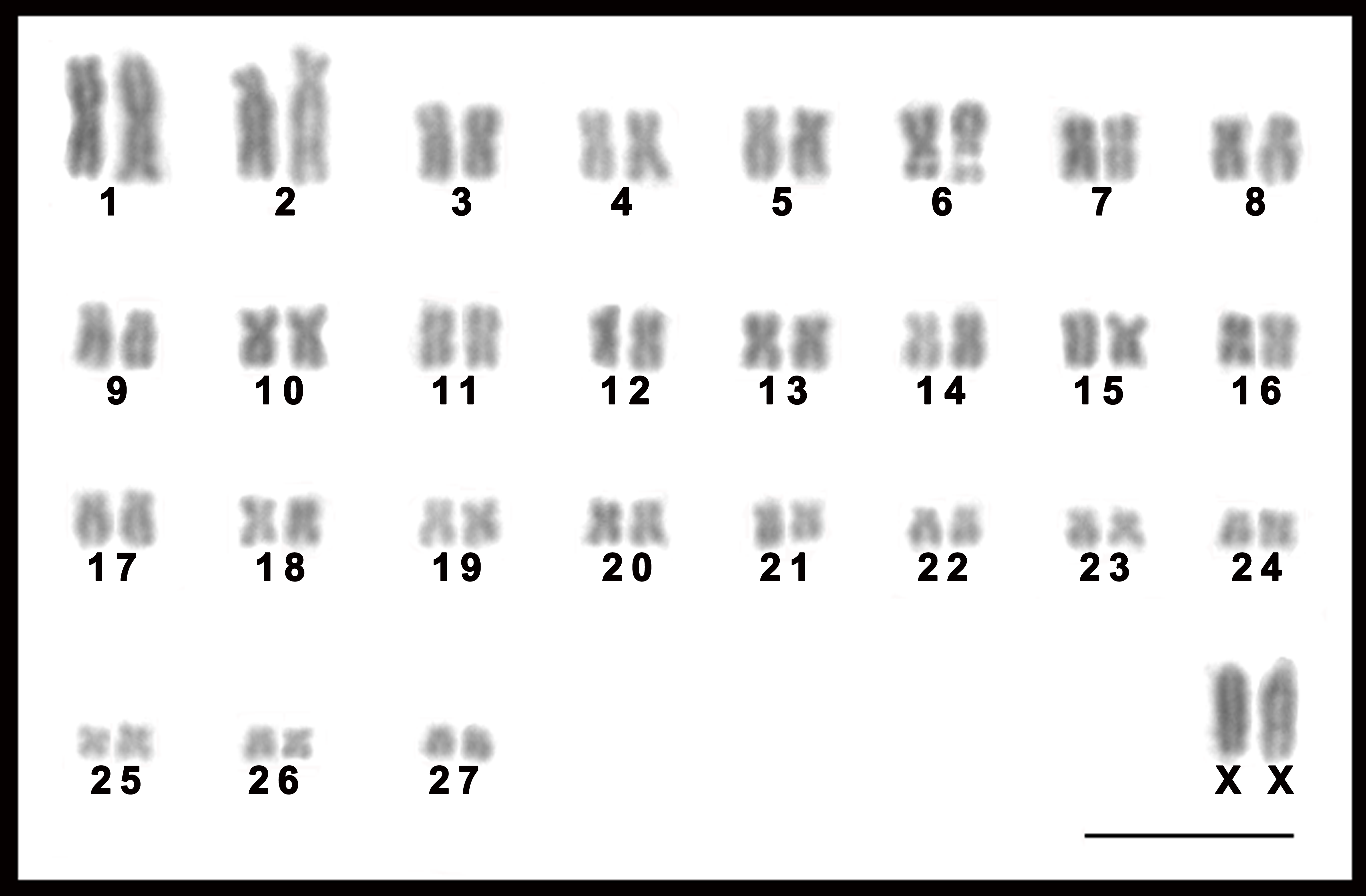

Supplement: Figure S4 — Karyotype of a female Trinomys setosus setosus (2n = 56, FN = 108), from Morro do Pilar, Minas Gerais state, after Giemsa staining. Scale bar = 10 µm. [file peerj-06-5316-s004.png]
