# Supplementary material for: Cytogenetic analyses in Trinomys (Echimyidae, Rodentia), with description of new karyotypes
Source: PeerJ. 2018 Jul 31;6:e5316. doi: 10.7717/peerj.5316 (PMC6074804; doi:10.7717/peerj.5316)
Supplement: Table S1 [file peerj-06-5316-s005.docx]

**Supplemental Table S1.** Morphological diagnosis of each specimen included in this study, compared to Trinomys moojeni and T. setosus setosus of other studies.

| **Species** | ***Trinomys moojeni*** | ***Trinomys setosus setosus*** | | | | ***Trinomys moojeni*** | ***Trinomys setosus setosus*** |
| --- | --- | --- | --- | --- | --- | --- | --- |
|  | MCN-M 2816 | MCN-M 2587 | MCN-M 3296 | MCN-M 3297* | UFMG 6024 | (n=7)^1,2^ | (n=28)^1,2,3^ |
| **CC** | 199 | 175 | 186 | 146 | 194 | 187 ± 18.2 | 204 ± 13.9 |
| **CA** | 174 | 195 | 195 | 152 | 191 | 179 ± 15.7 | 209 ± 16.1 |
| **Or** | 34 | 21 | 24 | 22 | 29 | 24 ± 1.5 | 27 ± 2 |
| **PP** | 51 | 48 | 44 | 41 | 50 | 44 ± 2.6 | 49 ± 2.7 |
| **MC** | 210 | 200 | 190 | 100 | 172 | - | 203.5 ± 33.5 |
| **Average length** | 50.11 | 48.66 | 49.15 | 71.86 | 47.93 | 50.2 ± 2.1 | 51.3 ± 2.4 |
| **Basilar length** | 35.25 | 34.24 | 33.93 | 28.23 | 33.83 | 35.5 ± 1.9 | 36.7 ± 1.8 |
| **Zygomatic width** | 24.99 | 24.4 | 25.42 | 22.64 | 24.64 | 25.5 ± 0.7 | 25.2 ± 1 |
| **Shape of incisive foramen** | Oval | Fusiform | Fusiform | Fusiform | Oval | Oval | Most fusiform |
| **Incisive foramen septum** | Incomplete, formed almost exclusively by premaxillae | Complete, with vomer participation | Complete, with vomer participation | Complete, with vomer participation | Complete, with vomer participation | Incomplete, formed almost exclusively by premaxillae | Complete, with vomer participation |
| **Post-orbital process** | Formed by squamosal and jugal | Formed exclusively by jugal | Formed exclusively by jugal | Formed exclusively by jugal | Formed exclusively by jugal | Formed by squamosal and jugal | Formed exclusively by jugal |
| **Bullae** | Small and smooth | Rounded and inflated | Rounded and inflated | Rounded and inflated | Rounded and inflated | Small and smooth | Rounded and inflated |
| **Brushy tail tip** | Poorly developed | Poorly developed | Well developed | Well developed | Well developed | Poorly developed | Present and mostly developed |
| **Dorsal aristiforms** | 0.73 | 0.66 | 0.74 | - | 0.72 | 0.6 | 0.7 |

*young specimen; CC – head and body; CA – tail length; Or – ear length; PP – hind foot; MC – body mass; MCN-M – Museu de Ciências Naturais – Pontifícia Universidade Católica (PUC, Minas Gerais, Brazil); UFMG – Centro de Coleções Taxonômicas – Universidade Federal de Minas Gerais (CCT-UFMG, Minas Gerais, Brazil); ^1^Bonvicino, Oliveira & D’Andrea (2008); ^2^Pessôa et al. (2015); ^3^Dalapicolla & Leite (2015).
