# Supplementary material for: Cytogenetic analyses in Trinomys (Echimyidae, Rodentia), with description of new karyotypes
Source: PeerJ. 2018 Jul 31;6:e5316. doi: 10.7717/peerj.5316 (PMC6074804; doi:10.7717/peerj.5316)
Supplement: Supplemental Information 1 [file peerj-06-5316-s006.docx]

**Supplemental Material**

**Part A**

**References for spatial data downloaded from IUCN for each *Trinomys* species:**

NatureServe and IUCN (International Union for Conservation of Nature) 2018. *Trinomys albispinus*. The IUCN Red List of Threatened Species. Version 2017-3 http://www.iucnredlist.org.

Downloaded on 26 April 2018.

NatureServe and IUCN (International Union for Conservation of Nature) 2018. *Trinomys dimidiatus*. The IUCN Red List of Threatened Species. Version 2017-3 http://www.iucnredlist.org.

Downloaded on 26 April 2018.

NatureServe and IUCN (International Union for Conservation of Nature) 2018. *Trinomys eliasi*. The IUCN Red List of Threatened Species. Version 2017-3 http://www.iucnredlist.org. Downloaded on 26 April 2018.

NatureServe and IUCN (International Union for Conservation of Nature) 2018. *Trinomys gratiosus*. The IUCN Red List of Threatened Species. Version 2017-3 http://www.iucnredlist.org.

Downloaded on 26 April 2018.

NatureServe and IUCN (International Union for Conservation of Nature) 2018. *Trinomys iheringi*. The IUCN Red List of Threatened Species. Version 2017-3 http://www.iucnredlist.org. Downloaded on 26 April 2018.

NatureServe and IUCN (International Union for Conservation of Nature) 2018. *Trinomys mirapitanga*. The IUCN Red List of Threatened Species. Version 2017-3 http://www.iucnredlist.org. Downloaded on 26 April 2018.

NatureServe and IUCN (International Union for Conservation of Nature) 2018. *Trinomys moojeni*. The IUCN Red List of Threatened Species. Version 2017-3 http://www.iucnredlist.org. Downloaded on 27 April 2018.

NatureServe and IUCN (International Union for Conservation of Nature) 2018. *Trinomys paratus*. The IUCN Red List of Threatened Species. Version 2017-3 http://www.iucnredlist.org. Downloaded on 27 April 2018.

NatureServe and IUCN (International Union for Conservation of Nature) 2018. *Trinomys setosus*. The IUCN Red List of Threatened Species. Version 2017-3 http://www.iucnredlist.org. Downloaded on 27 April 2018.

NatureServe and IUCN (International Union for Conservation of Nature) 2018. *Trinomys yonenagae*. The IUCN Red List of Threatened Species. Version 2017-3 http://www.iucnredlist.org.

Downloaded on 27 April 2018.

**Part B**

The mitochondrial cytochrome *b* GenBank accession numbers of *Trinomys* and outgroup included in this study. Underlined accession numbers, followed by the individual deposit numbers, refer to sequences generated in this study.

*Trinomys albispinus*: AF194282.1, EU313251.1, KM014008.1

*Trinomys dimidiatus*: AF194296.1, AF194297.1, AF194298.1, AF194299.1, AF194300.1, AF194301.1, AF194302.1, AF194303.1, AF194304.1, AF194305.1, U35167.1, U35168.1, U35169.1, U35170.1

*Trinomys eliasi*: AF194290.1, KF562079.1, KF562080.1, KF562081.1, KF562082.1, KF562083.1, KF562084.1, KF562085.1, KF562086.1, KF562087.1, KF562088.1, KF562089.1, KF562090.1, KF562091.1, KF562092.1, KF562093.1, KJ707244.1, KJ707245.1, KJ707246.1, KJ707247.1, U35166.1

*Trinomys gratiosus*: AF194278.1, AF194279.1, AF194280.1, AF194281.1, AF194327.1, AF194328.1, AF194329.1, AF194330.1, KJ707248.1

*Trinomys iheringi*: AF194306.1, AF194307.1, AF194308.1, AF194309.1, AF194310.1, AF194311.1, AF194312.1, AF194313.1, AF194314.1, AF194315.1, AF194316.1, AF194317.1, AF194318.1, AF194319.1, AF194320.1, AF194321.1, AF194322.1, AF194323.1, EU313254.1, EU313255.1, EU544664.1, U35171.1

*Trinomys moojeni*: KF562097.1, KX650080.1 (MCN-M2816)

*Trinomys paratus*: AF194291.1, AF194292.1, AF194293.1, AF194294.1, KF562094.1, U35165.1

*Trinomys setosus*: AF194283.1, AF194284.1, AF194285.1, AF194286.1, AF194287.1, AF194289.1, AF422923.1, AF422924.1, KF562095.1, KF562096.1, KX655539.1 (UFMG 6024), MG214347 (MCN-M 3296), MG214348 (MCN-M 3297), and MG214349 (MCN-M 2587)

*Trinomys yonenagae*: AF194295.1

*Euryzygomatomys spinosus*: EU544667
