# Supplementary material for: Cytogenetic analyses in Trinomys (Echimyidae, Rodentia), with description of new karyotypes
Source: PeerJ. 2018 Jul 31;6:e5316. doi: 10.7717/peerj.5316 (PMC6074804; doi:10.7717/peerj.5316)
Supplement: Supplemental Information 2 [file peerj-06-5316-s007.docx]

GenBank: MG214347

>Trinomys setosus_MCN-M 3296

acgtatctatgagcggtagctattactaagaagagtaagatgataccaatgtttcaggtttctataaaggtgtaggatccataatagattcctcgtccgatgtgaaaataaaggagaatgaaaaatatagaagctccgttggcatgtgcataacgaattattcaaccgtagtttacgtctcggcaaatgtgtgttacagatgaaaaggctgttattgtgtctgcagtgtagtgtatggctaggaataagcctgtaataatttgaagtgtaaggcatacacccaataaggaaccgaaatttcaccatgctgagatgttagatggtgctggtaaatcaatgaatgagtgattgatgattttaataagagggtgtgattttcggatgttggtcattaaagttcttatagttgaattacaacgatggtttttcat

GenBank: MG214348

>Trinomys setosus_MCN-M 3297

tattctatgaggcgggtagctattactaagaagagtaagatgataccaatgtttcaggtttctataaaggtgtaggatccataatagattcctcgtccgatgtgaaaataaaggagaatgaaaaatatagaagctccgttggcatgtgcataacgaattattcaaccgtagtttacgtctcggcaaatgtgtgttacagatgaaaaggctgttattgtgtctgcagtgtagtgtatggctaggaataagcctgtaataatttgaagtgtaaggcatacacccaataaggaaccgaaatttcaccatgctgagatgttagatggtgctggtaaatcaatgaatgagtgattgatgattttaataagagggtgtgattttcggatgttggtcattaaagttcttatagttgaattacaacgatggtttttc

GenBank: MG214349

>Trinomys setosus_MCN-M 2587

gtatcctatgagcggtagctattactaagaagagtaggatgacgccaatatttcaggtttcctataaaggtgtaggatccataatagattcctcgtccgatgtgaaagtaaaggagaatgaaaaatatagaagctccgttggcatgtgcataacgaattattcaaccgtagttcacgtctcggcaaatatgtgttactgatgaaaaggctgttattgtatctgcagtataatgtatggctaggaataagcctgtaataatttgaagtgtgaggcatacacccaataaagaaccaaaatttcatcatgctgagatgttagatggtgctggtaaatcaatgaatgagtgattgatgattttaataagagggtgtgattttcgaatattggtcattaaagttcttatagttgaattacaacgatggtttttcata
